# Weighted Correlation Network Analysis Reveals CDK2 as a Regulator of a Ubiquitous Environmental Toxin-Induced Cell-Cycle Arrest

**DOI:** 10.3390/cells9010143

**Published:** 2020-01-07

**Authors:** Virginie Dubourg, Alexander Nolze, Michael Kopf, Michael Gekle, Gerald Schwerdt

**Affiliations:** Julius-Bernstein-Institute for Physiology, Martin-Luther-University Halle-Wittenberg, 06112 Halle (Saale), Germany; alexander.nolze@medizin.uni-halle.de (A.N.); michael.kopf@medizin.uni-halle.de (M.K.); michael.gekle@uk-halle.de (M.G.); gerald.schwerdt@medizin.uni-halle.de (G.S.)

**Keywords:** transcriptomics, human kidney, nephrotoxicity, ochratoxin A, WGCNA, cell cycle, CDK2, CDKN1A/p21

## Abstract

Environmental food contaminants constitute a threat to human health. For instance, the globally spread mycotoxin Ochratoxin A (OTA) contributes to chronic kidney damage by affecting proximal tubule cells via unknown mechanisms. We applied a top-down approach to identify relevant toxicological mechanisms of OTA using RNA-sequencing followed by in-depth bioinformatics analysis and experimental validation. Differential expression analyses revealed that OTA led to the regulation of gene expression in kidney human cell lines, including for genes enriched in cell cycle-related pathways, and OTA-induced gap 1 and 2 (G1 and G2) cell-cycle arrests were observed. Weighted correlation network analysis highlighted cyclin dependent kinase 2 (CDK2) as a putative key regulator of this effect. CDK2 was downregulated by OTA exposure, and its overexpression partially blocked the OTA-induced G1 but not G2 cell-cycle arrest. We, therefore, propose CDK2 as one of the key regulators of the G1 cell-cycle arrest induced by low nanomolar concentrations of OTA.

## 1. Introduction

Chronic exposure to environmental food contaminants like mycotoxins represents a hidden threat to human health. Unfortunately, in many cases, the underlying mechanisms are insufficiently understood and difficult to investigate experimentally. Sometimes a deductive approach is successful; however, in many cases, the mechanisms at the cellular level remain elusive, making risk assessment and the development of therapeutic strategies difficult. Because chronic exposure to toxic agents will result in alterations of the transcriptome, an alternative approach consists of the in-depth bioinformatics analysis of transcriptomics data combined with pathway analysis of differential RNA expression to identify upstream pathways or canonical regulators. We applied this approach for the mycotoxin Ochratoxin A (OTA), a widespread environmental food contaminant known to cause impaired renal function.

OTA is produced by *Aspergillus* and *Penicillium* species and is mainly found in cereals but also in coffee, wine, beer, cacao, or pork meat [1]. Due to its high incidence in foodstuffs even after processing, exposure risk for humans is very high. OTA concentrations in blood average around 1 nM in populations who nourish on a normal diet [1,2,3] but it can increase (up to around 10 nM) in some regions where the daily intake is higher due to, e.g., geographic situation, as well as climatic, social, and economic conditions [3,4,5]. OTA concentrations can even get way above 10 nM in some pathological cases, as concentrations up to 160 nM were previously reported [4].

The kidney is the main target of OTA [6,7,8] due to the accumulation of the toxin in renal tissue by efficient proximal tubular transport [9]. Proximal tubule cells are, thus, exposed to the highest concentrations of OTA in the organism, which remain in the nanomolar range [9]. Despite this evidence, many published studies were based on experiments with higher OTA concentrations (micromolar range and even higher) and may, therefore, report unspecific toxicological reactions. We previously observed that nanomolar OTA triggers apoptosis [10], leads to gene expression dysregulation [11,12], influences signaling pathways [11,13], and can induce characteristic traits of interstitial nephritis in vitro [14]. However, the understanding of OTA nephrotoxicity following exposure to nanomolar concentrations is still limited. In the present study, we investigate the effect of OTA on the transcriptome of human kidney cells in an attempt to decipher the underlying mechanisms and to identify key actors in the latter. RNA-sequencing was performed on two human kidney cells lines exposed to 10 or 100 nM OTA, concentrations eventually found in the blood of people who nourish on a diet rich in OTA-contaminated food [3,4,5]. The results suggest that OTA leads to wide changes in the transcriptome of human kidney cells associated with cell cycle-, cell survival-, and DNA damage-related functions. OTA-induced cell-cycle dysregulation could be confirmed, and the results of weighted correlation network analysis brought up cyclin-dependent kinase 2 (CDK2) as one of the major regulators of this phenotype.

## 2. Materials and Methods

### 2.1. Cell Culture and Treatment with OTA

HK-2 (human (*Homo sapiens*) proximal tubule epithelial cell line) and HEK293-T (human (*Homo sapiens*) embryonic kidney cell line) were obtained from American Type Culture Collection (ATCC). Both cell lines were cultivated in Dulbecco’s modified Eagle’s medium (DMEM)/Ham’s F-12 medium supplemented with 10% fetal calf serum (FCS) at 37 °C, 5% CO_2_. They were incubated for 24 h in serum-free medium before treatment with 0, 10, or 100 nM OTA (final concentrations) for 48 h in serum-free medium. Serum free medium was used (1) to avoid the variability in between different FCS batches, and (2) because tubular fluid, with which proximal tubular cells are in contact with, contains only a low amount of proteins. For cell-cycle analysis, cells from these different conditions were additionally cultivated with 0 or 100 µg/L epidermal growth factor (EGF—final concentration).

### 2.2. RNA Isolation and Sequencing on Human Kidney Cells

Total RNA was isolated from HK-2 and HEK293-T cells using TRIzol Reagent (Life Technologies, Schwerte, Germany) following the manufacturer’s instructions. RNA precipitation with isopropanol was performed overnight at −20 °C, and the obtained pellet was washed twice with 75% ethanol. RNA concentration was measured using a NanoVue Plus spectrophotometer (GE Healthcare, Freiburg, Germany). Additional ethanol precipitation, quality control (with Bioanalyzer, Agilent Technologies, Waldbronn, Germany), complementary DNA (cDNA) library, preparation and RNA-sequencing were performed at the Core Unit DNA Technologies of the Faculty of Medicine, University Leipzig (Leipzig, Germany). Briefly, ribosomal RNAs (rRNA) were removed from 500 ng of total RNA using RiboMinus kit (Life Technologies) according to the manufacturer’s instructions. The rRNA-depleted RNA was fragmented (treatment with 200 nM Tris-acetate—pH 8.2, 500 mM potassium acetate, and 150 nM magnesium acetate followed by heating at 94 °C for 3 min) and precipitated (with 100% ethanol, ammonium acetate, glycogen). The fragmented RNA was used to generate cDNA libraries with indexed adapters (Illumina, Berlin, Germany). Then, 10 nM pools of up to 10 libraries were used for cluster generations (Illumina cBot). For the samples isolated from HK-2 and HEK293-T cells, paired-end sequencing (2 × 100 bp) was performed on Illumina HiSeq2000 and HiScanSQ sequencers, respectively.

### 2.3. Cell-Cycle Analysis with 5-Bromo-2-deoxyuridine (BrdU) Incorporation

OTA-influence on cell cycle was measure by BrdU-based flow cytometry. BrdU (5-bromo-2-deoxyuridine) was added to HK-2 and HEK293-T cells at a final concentration of 10 µM one hour prior to harvesting. Then, 1 × 10^6^ cells were washed with 1× phosphate-buffered saline (PBS—13.7 mM NaCl, 0.3 mM KCl, 0.8 mM Na_2_HPO_4_·2H_2_O, 0.2 mM KH_2_PO_4_—pH 7.4) by centrifugation (200× *g*, 5 min, 4 °C) and fixated with 75% ice-cold ethanol at −20 °C overnight. On the next day, after centrifugation (500× *g*, 5 min, 4 °C), the cells were incubated with 2 N HCl, 0.5% Triton X-100 (DNA denaturation) at room temperature for 30 min. Then, 0.1% Na_2_B_4_O_7_ (neutralization) was added directly to the samples before centrifugation. Cells were rinsed twice with 1× PBS. The pellet was resuspended in antibody solution (1× PBS, 0.5% Tween-20) with mouse anti-BrdU antibodies (diluted 1:5 in antibody solution—BD Biosciences, cat. no. 347580) and incubated for 1 h at 4 °C. Additional samples were incubated with mouse immunoglobulin G (IgG) isotope control antibodies (diluted 1:40 in antibody solution—BD Biosciences, cat. no. 349040). All samples were then washed with 1× PBS and incubated with fluorescein isothiocyanate (FITC)-conjugated anti-mouse antibodies (diluted 1:200 in antibody solution—Rockland Immunochemicals, cat. no. 610-102-040) for 2 h at 4 °C. After washing, cells were finally stained with PI (propidium iodide) diluted in 1× PBS at a final concentration of 50 µg/mL. RNase A (Qiagen, Hilden, Germany) was added at a final concentration of 0.2 mg/mL. Cells were incubated for 10 min at 4 °C before measurement with an LSR Fortessa flow cytometer (BD Biosciences). Living single cells only were considered for the analysis of the data (gate based on the forward scatter—FSC—the side scatter—SSC—and the PI-corresponding channel).

### 2.4. CDKN1A/p21 Knockdown

The expression of *CDKN1A/p21* was clamped down using specific small interfering RNA (siRNA) (Ambion, cat. no. 4390824, Darmstadt, Germany); scramble siRNAs (Ambion, cat. no. 4390843) were used as a negative control. HK-2 cells were cultivated in 6-cm dishes to reach 60%–80% confluence. The cells were transfected with a final concentration of 20 pmol of these siRNAs using Lipofectamine (ThermoFisher, Schwerte, Germany), following the instructions of the manufacturer. After 24 h of incubation with transfection reagent, the “pooled transfected” cells were transferred to 24-well plates where they were successively cultivated for 24 h in media with FCS, for 24 h in serum-free media, and for 24 h in serum-free media supplemented with 0, 10, or 100 nM OTA.

### 2.5. CDK2 Overexpression

HK-2 cells were cultivated in 6-cm dishes to reach between 60% and 80% confluency. Cells were transfected with a *CDK2* overexpression vector (Origene, cat. no. SC109060, Rockville, MD, USA) as follows: 2 µg of vector and 20 µL of PolyFect transfection reagent (Qiagen) were diluted in 150 µL of serum-free media, and the tubes were incubated for 10 min at room temperature; the medium was removed from the cell culture dishes and 1 mL of media with FCS was added; 500 µL of media with FCS was added to the transfection complexes, and the whole volume (672 µL) was directly transferred to the cells. Additionally, some cells were incubated with PolyFect transfection reagent but without *CDK2* overexpression vector. All cells were transferred 24 h later in 24-well plates and, from the next day on, they were cultivated successively for 24 h with serum-free media and 24 h with serum-free media supplemented with 0 or 100 nM OTA before cell-cycle analysis and protein isolation.

### 2.6. Detection of CDKN1A/p21, CDK2, and E2F

Total RNA for RT-qPCR was isolated using InviTrap spin tissue RNA mini kit (Invitek Molecular GmbH, Berlin, Germany) following the manufacturer’s instructions. DNA contamination was removed (DNAse I, New England Biolabs), and reverse transcription (RT) was performed using random primers and SuperScript II reverse transcriptase (Invitrogen, Life Technologies) according to the manufacturer’s instructions. Then, 1 µL of the obtained cDNA was used in RT-qPCR (AriaMx Real-Time PCR System, Agilent Technologies) to confirm differential expression of *CDKN1A/p21* (sense: 5′ ACTTCGACTTTGTCACCGAG 3′, antisense: 5′ GTCCACATGGTCTTCCTCTG 3′), *CDK2* (sense: 5′ ATTCATGGATGCCTCTGCTC 3′, antisense: 5′ TTTAAGGTCTCGGTGGAGGA 3′), *E2F1* (sense: 5′ GCCAAGAAGTCCAAGAACCA 3′, antisense: 5′ TCTGCAATGCTACGAAGGTC 3′), and *E2F4* (sense: 5′ ATAGTCCTCAGCTCACTCCC 3′, antisense: 5′ GTCCTTGCTATCAGTCCCAG 3′). The results were normalized with the house keeping genes *EEF2* (sense: 5′ GGAGTCGGGAGAGCATATCA 3′, antisense: 5′ GGGTCAGATTTCTTGATGGG 3′) and *RPS17* (sense: 5′ TCAGCCTTGGATCAGGAGAT 3′, antisense: 5′ CATCCCAACTGTAGGCTGAG 3′). The following thermal profile was used for all targets: 10 min at 95 °C, followed by 40 amplification cycles (10 s at 95 °C, 20 s at 60 °C and 20 s at 72 °C), 5 min at 72 °C, and by melting curve determination.

For protein expression level determination, cells were lysed with Cell Signaling Technology (CST) lysis buffer, and protein amount was determined with Bradford assay [15]. Equal amounts of proteins were denatured with 6× Laemmli buffer (Table S7, Supplementary Materials), separated by 12% SDS-PAGE, and transferred to nitrocellulose membranes. CDKN1A/p21 (Cell Signaling Technology, cat. no. 2946, Frankfurt, Germany), CDK2 (Cell Signaling Technology, cat. no. 2546), and E2F4 (R&D, cat. no. AF5139) were detected using IRDye-coupled fluorescent secondary antibodies (LI-COR Biosciences, Bad Homburg, Germany) and an Odyssey imaging system from LI-COR Biosciences. E2F1 (R&D, cat. no. AF4825) was detected by chemiluminescence. Estimation of protein amount was made (based on the density of protein bands) with the Quantity One software (version 4.6.9, Bio-Rad, Feldkirchen, Germany).

### 2.7. Measure of Energy-Related Parameters

To estimate changes in mitochondrial membrane potential, HK-2 cells were exposed to 0, 10, or 100 nM OTA for 48 h and were stained using JC-10 dye (Sigma-Aldrich, München, Germany) following the manufacturer’s instructions. A Cytation 3 Image Reader (BioTek, Berlin, Germany) was used for signal detection.

Additionally, ATP content of HK-2 cells was measured after 48 h incubation with 0, 1, 10, or 100 nM OTA using an ATP Bioluminescence Assay Kit HS II (Roche, Basel, Switzerland) following the manufacturer’s instructions. A TriStar LB941 Luminometer (Berthold Technologies, Bad Wildbad, Germany) was used for detection. Protein concentrations were determined after cell lysis using bicinchoninic acid (BCA) in order to normalize the amount of ATP obtained for each cell-culture well.

### 2.8. RNA-Sequencing Data Analysis

Quality controls (fastQC, v0.11.3, https://www.bioinformatics.babraham.ac.uk/projects/fastqc/) were performed on the raw RNA-sequencing data and following adapter sequence clipping with Cutadapt (v1.8.1, using the parameters q 20, O 7, m 0) [16]. Alignments to the human genome hg38 and counting were performed using tophat (2.0.14, with library-type fr-unstranded, –b2-N 1) [17] and featureCounts (1.4.6, with -p -M -t exon -g gene_id) [18], respectively.

### 2.9. Differential Expression Analysis

Differential expression analysis was performed using R packages EdgeR (3.20.8) [19] and DESeq2 (1.20.0) [20] from Bioconductor (https://www.bioconductor.org/). For the analysis with EdgeR, the counts were normalized using the “trimmed mean of M values” or TMM method. During DESeq2 analysis, genes were filtered for those with at least one normalized count in more than three replicates before normalization with the “regularized log transformation” (rlog) function. A false discovery rate (FDR) of 0.01 was used to determine if genes were significantly regulated. Only the genes that were found regulated with both tools (overlap of the analysis outputs) were considered for further analysis steps. Additionally, genes with abs(log2 Fold Change) ≥ 1 and with more than 10 FPM (fragments per million) on average (in control samples when downregulated and in treated samples when upregulated) were filtered. Annotation of the analyzed genes was made with Ensembl BiomaRt v93 (R package biomaRt v2.36.1 [21]).

### 2.10. Weighted Correlation Network Analysis

Correlation network analysis was performed using the R package WGCNA (1.63) [22]. The construction of the network was based on the RNA-sequencing data obtained from HK-2 cells (18 samples, incubated with 0, 10, or 100 nM OTA). Only genes with counts > log2(150) after “variance stabilizing transformation” (from DESeq2 R package) in at least six samples (corresponding to the smallest sample size of a given condition) were kept. A soft power (β) of 13 was applied for network construction. No dissimilarity threshold was applied. Correlation coefficients between the module Eigengenes and traits (e.g., treatment with OTA, cell type) were calculated using Pearson’s method.

### 2.11. Functional Analysis

Ingenuity Pathway Analysis (IPA) software (Qiagen) was used for functional analysis (Canonical Pathways, Upstream Regulator and Downstream Effects Analyses) on the lists of OTA-regulated genes (results of the differential expression analyses) and of genes belonging to network modules found to be highly correlated (abs(corr) ≥ 0.75) with OTA treatment (results of weighted correlation network analysis). Their Ensembl identifiers were mapped to networks available in the software database. For the canonical pathway analysis, enriched pathways were ranked according to how relevant they were for the genes provided as input. Multiple testing was performed using the Benjamini–Hochberg (B–H) procedure.

### 2.12. Transcription Factor Binding Site Enrichment

Transcription factor binding site enrichment was performed using g:profiler (https://biit.cs.ut.ee/gprofiler/) on the lists of genes found to be significantly regulated by 100 nM OTA in HK-2 and in HEK293-T cells. An FDR of 0.01 was used after multiple testing.

### 2.13. Evaluation of Molecular Biology Parameter Measurements

All the data are presented as means ± standard deviation for each group. A two-tailed *t*-test was performed for each experiment and a *p*-value threshold of 0.05 was used for significance determination. Statistical tests were performed with ggpubr R package (https://cran.r-project.org/package=ggpubr). *N* indicates the numbers of biological replicates (cell line passages) in the figure legends, and *n* indicates the total number of samples (petri dishes).

For the results of the BrdU-based cell-cycle analysis, multiple testing (Benjamini–Hochberg procedure) was applied to the results. Outlier exclusion was performed using a chi-squared test from the R package outliers (https://cran.r-project.org/package=outliers).

### 2.14. Data Availability

Raw RNA-sequencing data and raw counts are publicly available on the GEO (Gene Expression Omnibus) database, GEO identifier (ID): GSE133831.

## 3. Results

### 3.1. OTA Exposure Induces Wide Changes in Gene Expression in Human Kidney Cell Lines, Including for Genes Enriched in Cell Cycle-Related Pathways

RNA-sequencing was performed on total RNA isolated from HK-2 and HEK293-T cells incubated with low concentrations of OTA (0, 10 or 100 nM) in order to identify putative effects of this mycotoxin on the transcriptome of human kidney cells. Differential expression analyses were performed with EdgeR [19] and DESeq2 [20] to compare 0 vs. 100 nM OTA-treated HEK293-T cells, as well as 0 vs. 10 nM OTA- and 0 vs. 100 nM OTA-treated HK-2 cells. Because lowly expressed genes are unlikely to present expression differences due to high dispersion when performing analyses with DESeq2, the genes not detected (with no normalized counts) in at least three samples were filtered out before proceeding. Only genes with adjusted *p*-values (FDR) ≤ 0.01 were considered. The analyses with DESeq2 led to the identification of higher numbers of differentially expressed genes than with EdgeR, but most regulated genes were found by both tools, and the respective log2 fold changes showed a high and linear correlation (Figure S1, Supplementary Materials). Therefore, only the genes found to be differentially expressed with both tools were considered as OTA-regulated and were used for further analysis. Additionally, genes with low variation (abs(log2 fold change) < 1) and with low counts (FPM < 10) were filtered out.

**Figure 1:**
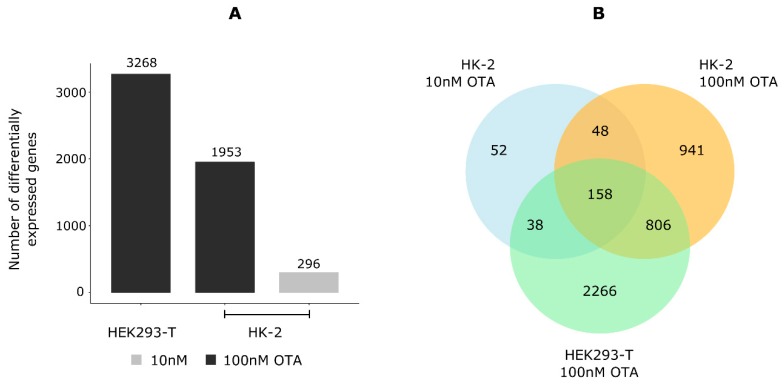
Ochratoxin A (OTA) led to differential expression of genes in human kidney cells. (**A**) Genes with abs(log2 fold change) ≥ 1 and fragments per million (FPM) ≥ 10 on average (in control samples when downregulated and in treatment samples when upregulated) were filtered from the list of genes significantly regulated by OTA (false discovery rate (FDR) 0.01) in the output of both EdgeR and DESeq2. (*N* = 3–4) (**B**) The outputs of the differential expression analyses were crossed, and 158 genes were found constitutively regulated by OTA. See also Figures S1–S3 and Table S2 (Supplementary Materials).

Following this principle, 3268, 296, and 1953 genes were identified as significantly regulated in HEK293-T cells after incubation with 100 nM OTA, and in HK-2 cells after incubation with 10 and 100 nM OTA, respectively (Figure 1A, Table S1, Supplementary Materials). More than 85% of the regulated genes were in each case coding for proteins (Figure S2, Supplementary Materials). The differential expression of genes randomly picked through these lists could be confirmed by RT-qPCR (data not shown). When checking for the overlap of these datasets, 158 genes appeared to be constitutively regulated by OTA in all datasets, including with 10 nM OTA (Figure 1B). Here, 206 genes were differentially expressed at both OTA concentrations in HK-2 cells, and their expression levels showed a high linear correlation (*R* = 0.96) (Figure S3A, Supplementary Materials). An additional differential expression analysis was performed to identify a putative dose effect (10 nM OTA- vs. 100 nM OTA-treated HK-2 cells). Out of 206 genes regulated by the two types of treatment, 17 genes (8%) were found to be regulated in a concentration-dependent manner (Table S2, Supplementary Materials). Finally, when comparing the results of the differential expression analyses for HEK293-T and HK-2 after incubation with 100 nM OTA, 964 genes were found differentially expressed irrespective of the cell type (Figure 1B). The expression levels of these genes showed a linear correlation (*R* = 0.9) even though OTA appeared to induce opposite regulation for 20 of them (Figure S3B, Supplementary Materials).

Pathway enrichment analysis was performed to identify functions in which the OTA-regulated genes may be involved. However, a large number of genes were regulated in a cell-type-specific manner (Figure 1B) and we were interested in identifying a more general effect of the mycotoxin on human kidney cells. This analysis step was, therefore, performed with the genes significantly regulated in both HEK293-T and HK-2 cells when treated with 100 nM OTA. The 20 genes that were regulated in the opposite direction in both cell lines were excluded, lowering the number of considered genes to 944. The output of this analysis showed that genes were significantly enriched (adjusted *p*-value ≤ 0.05) in DNA damage- and cell-cycle control-related pathways (Table 1), including in some that were predicted to be downregulated (*Z*-score < 0).

### 3.2. OTA Leads to Cell-Cycle Arrest in Human Kidney Cell Lines

**Figure 2:**
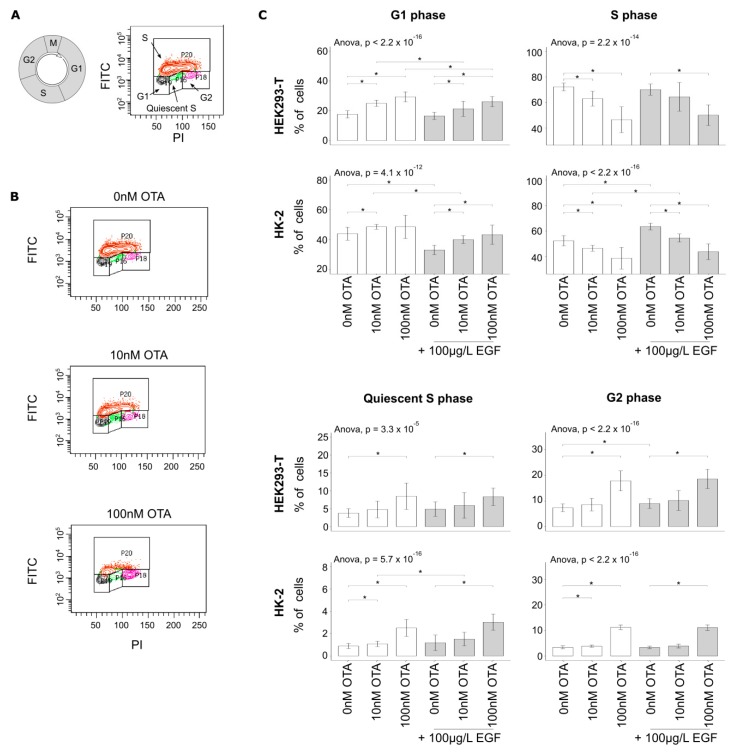
Gap 1 and 2 (G1 and G2) cell-cycle arrests were induced by different concentrations of OTA. (**A**) Illustration of the strategy used to determine the percentages of cells in each population. (**B**) Example of OTA-induced changes in the distribution of cell populations (here in HEK293-T cells). Anti-5-bromo-2-deoxyuridine (BrdU) antibodies were coupled with fluorescein isothiocyanate (FITC). (**C**) Cells were exposed to different concentrations of OTA and epidermal growth factor (EGF). We excluded the samples that were not stained properly (visual selection) and the outliers (statistics-based selection). Pairwise *t*-test and multi-testing were performed (mean ± SD, * *p* < 0.05, *N* = 5, *n* = 11–15). See also Figure S5 (Supplementary Materials).

We observed that OTA exposure led to an increase of the number of cells in gap 1 (G1) phase at low concentrations (10 nM), also in presence of the growth factor EGF (Figure 2A–C). This suggests that OTA induces a G1 cell-cycle arrest in human kidney cell lines at 10 nM. The decrease in the number of cells in the synthesis (S) phase supports this suggestion. Additionally, the higher concentrations of OTA (100 nM) led to an increase in the number of cells in quiescent S phase (cells which entered the S phase but stopped to synthetize DNA before reaching the G2 phase) and an increase of cells in G2 phase. Similar results were obtained with PI staining which excluded an effect of the OTA genotoxicity on these results based on BrdU incorporation. Thus, these results imply that OTA can lead to the interruption of DNA synthesis and to G2 cell-cycle arrest but at higher concentrations only. OTA was previously associated with dysregulation in energy homeostasis which could result in such cell-cycle disruption. However, no changes in ATP and mitochondrial potential were observed after OTA exposure (Figure S4, Supplementary Materials). We, therefore, suggest that this phenotype is due to changes in the expression and activity of a cell-cycle key regulator.

### 3.3. CDKN1A/p21 Is Not Responsible for the OTA-Induced Cell-Cycle Dysregulation

Cell-cycle regulation involves various proteins that regulate the progression from one phase to another. CDKN1A/p21 is one of the major inhibitors of the G1/S phase checkpoint but is also involved in the maintenance of the G2/mitosis (M) checkpoint [23]. The differential expression analyses revealed that the gene *CDKN1A/p21* was upregulated from 10 nM OTA (Figure S5A, Supplementary Materials), which could be confirmed by RT-qPCR and by Western blot (Figure S5B,C, Supplementary Materials). To test the importance of *CDKN1A*/*p21*-upregulation for cell-cycle arrest, its expression was knocked down by siRNA (Figure S5D). However, subsequent cell-cycle analysis revealed that the knockdown of *CDKN1A/p21* did not prevent OTA-induced cell-cycle dysregulation (Figure S5E, Supplementary Materials). Furthermore, the main activator of *CDKN1A/p21* transcription, the transcription factor p53, was not upregulated by OTA-exposure. Differential expression analysis even showed it to be downregulated by exposure to 100 nM OTA in HK-2 cells (log2 fold change = −1.31, B–H *p*-value = 4.47 × 10^−11^), which could be confirmed by RT-qPCR (Figure S5F, Supplementary Materials). These results suggest that CDKN1A/p21 and the p53/p21 pathway are not the major regulators of this OTA-induced phenotype.

### 3.4. Weighted Correlation Network Analysis Was Used to Identify Putative Key Drivers of the OTA-Induced Phenotype

To avoid accumulating false candidates, a more elaborated strategy—weighted correlation network analysis—was developed for the selection of potential regulators of the OTA-induced phenotype.

The network was constructed using the WGCNA R package [22], which assigns a numerical value to the connection between each considered genes. This value is called “adjacency” and depends on the correlation between two genes, with adj(*X*,*Y*) = ((corr(*X*,*Y*) + 1)/2)*^β^*. A network needs to satisfy the scale-free topology criterion (*R*^2^ > 0.80) to be considered as statistically reliable [22], and the selection of the soft-power *β* is a critical step to achieve this. However, no value leading to such a network was found while screening *β* values if all the samples available in our datasets were considered (Figure S6A, Supplementary Materials). Such a phenomenon can occur when a strong biological variable leads to high correlation between groups of samples. Suspecting that the simultaneous study of two different cell lines could be the reason why an adequate soft-power was lacking, a second network was constructed considering HK-2 cell samples only. A soft-power *β* of 13 permitted in that case to reach a scale-free topology (*R*^2^ = 0.85, Figure S6B). This result suggests that the biological variable “cell type” was indeed responsible for the difficulties of obtaining an optimal soft-power *β* when considering all samples. The high number of differentially expressed genes when comparing HK-2 and HEK293-T cells supports this idea (analysis performed on samples not exposed to OTA only—Table S1, Supplementary Materials). The network was, thus, constructed on RNA-sequencing data obtained from HK-2 cells exposed to 0, 10, or 100 nM OTA only.

**Figure 3:**
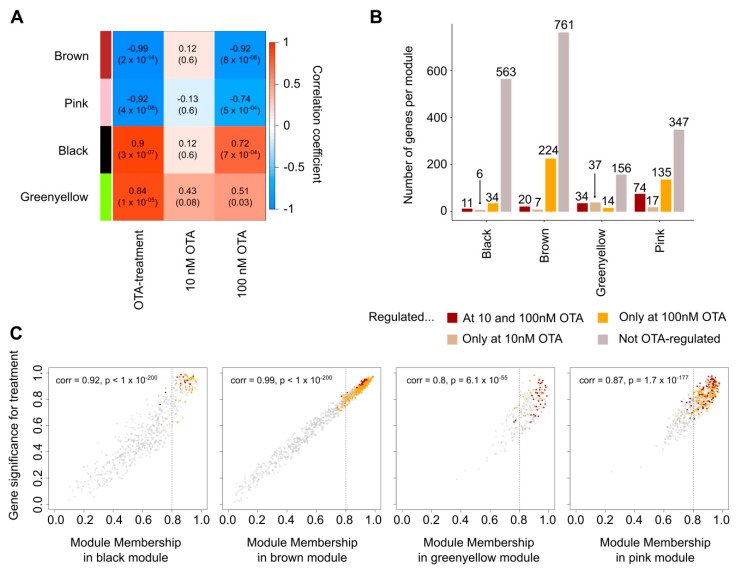
Weighted correlation network analysis was used to identify groups of genes putatively involved in OTA-induced cell-cycle dysregulation. (**A**) The heatmap represents the modules with high correlation between their Eigengenes and the “OTA-treatment” trait (abs(corr) ≥ 0.75) and with abs(corr_treatment_) > abs(corr_100nM OTA_) (to avoid unspecific effects due to too high OTA concentrations). (**B**) The numbers of genes found to be regulated by OTA (by differential expression analyses) in modules from (**A**) are displayed. No distinction between the cell types is made in this figure (overlap of the analyses output). (**C**) The gene significance for the trait “OTA treatment” and the module membership were calculated for each of the modules presented in (**A**). Each dot corresponds to a gene. The dashed vertical line corresponds to the threshold (0.8) used for selecting hub-like genes. See also Figures S6 and S7 (Supplementary Materials).

This strategy had initially the advantage of considering all annotated genes (found to be regulated by OTA in the first phase of our study or not). Nevertheless, lowly expressed genes were excluded in order to avoid spurious correlations during the construction of the network, lowering the number of considered genes to 9489. The network constructed on these genes led to the identification of 15 modules comprising genes with similar expression patterns (Table S3, Supplementary Materials). Subsequently, we filtered the modules whose Eigengene (theoretical module representative gene, noted E) [24] were highly correlated with OTA treatment (abs(corr_(E,treatment)_) ≥ 0.75, *p* < 0.05) but with abs(corr_(E,treatment)_) > abs(corr_(E,100nM OTA)_) (to avoid unspecific effects due to too high OTA concentrations). The results for the four remaining modules out of the 15 initially identified ones are presented in a simplified heatmap in Figure 3A (complete data in Figure S7, Supplementary Materials). The colors automatically assigned by WGCNA are used to refer to these modules. “Downstream effects analysis” was performed with IPA Software for each module in order to identify which functions may be regulated by the genes they comprised. Out of the four considered modules, three (*brown*, *green-yellow*, and *pink*) contained genes significantly enriched in cell cycle-related functions (Table 2) and more precisely in, e.g., “cell-cycle progression”, “arrest in G2 phase”, “delay in initiation of G1/S phase transition”, and “arrest in mitosis” (Table S4, Supplementary Materials).

Intramodular hub-genes (genes with high intramodular connectivity) of a module are supposedly more biologically relevant and are putative regulators of the functions fulfilled by the genes comprised in the same module [25]. To identify such genes, gene significance (GS) for the trait “OTA treatment” and module membership (MM) of each gene belonging to the selected modules were calculated (Figure 3C). Roughly, the former corresponds to the biological significance of the genes for a given trait, while the MM corresponds to the correlation of a gene with the module Eigengene (e.g., the MM *kME* of a gene *i* in the *brown* module is *kME_i_* = corr(*i*, E*^brown^*), with E*^brown^* the module Eigengene of this module) [22]. This was used as an estimate of the intramodular connectivity since the MM is easier to compute and both values are highly correlated in single networks [26]. Therefore, genes with high MM (*kME* ≥ 0.80) were considered as intramodular hub-like genes. Cautious of keeping focus on the genes that may be involved in an OTA-induced phenotype in both cell lines, and despite the fact that the network was based only on the data from HK-2 cells, we integrated the results of the differential expression analyses by looking for genes that were constitutively regulated by OTA. The integration of these results to the weighted correlation network analysis revealed that the modules with abs(corr_(E,treatment)_) ≥ 0.75 contained genes significantly regulated by OTA in both HEK293-T and HK-2 cells (Figure 3B), and that some of the latter were hub-like genes according to the definition given above (Figure 3C).

We focused on the *brown* module which comprised genes with high correlation (corr = 0.99) between their GS for “OTA treatment” and their MM (Figure 3C). This suggested that the hub-like genes of this module were more likely to be relevant for the OTA treatment biological trait. The well-described cell-cycle key regulator *CDK2* (ENSG00000123374, *kME* = 0.93) was both classified as a hub-like gene of this module and found to be significantly regulated by OTA.

### 3.5. CDK2 Is Involved in the OTA-Induced Dysregulation of the Cell Cycle

**Figure 4:**
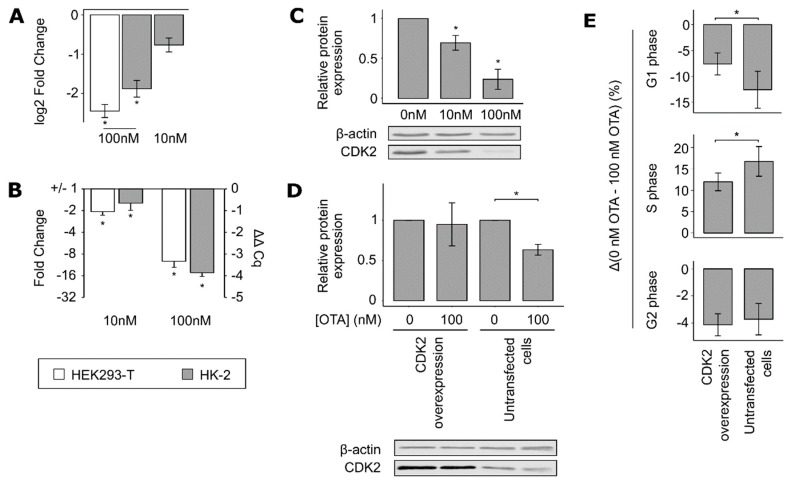
Overexpression of cyclin-dependent kinase 2 (CDK2) partially restores the OTA-induced G1/synthesis (S) cell-cycle arrest. (**A**) *CDK2* was significantly downregulated following exposure to 100 nM OTA according to the RNA-sequencing data. Additionally, a tendency to downregulation at 10 nM OTA was observed without reaching the FDR threshold of 0.01. This OTA-induced regulation of *CDK2* was confirmed by RT-qPCR (**B**) and Western blot (**C**) from 10 nM OTA onward (mean ± SD, *N* = 3, * *p* < 0.05 when comparing to the samples treated with 0 nM OTA). (**D**) After 24 h of incubation with 100 nM OTA, *CDK2* was significantly downregulated in untransfected cells but not in cells transfected with a *CDK2* overexpression vector (mean ± SD, * *p* < 0.05, *N* = 4). (**E**) Cell-cycle analysis was performed on HK-2 cells transfected with *CDK2* overexpression vector or just treated with transfection reagent. The OTA effect is here represented as the difference between the percentages of cells in each phase when treated with 0 and 100 nM OTA (mean ± SD, * *p* < 0.05, *N* = 4, *n* = 7–8).

Based on the results of the weighted correlation network analysis, CDK2 was considered as a putative regulator of the OTA-induced changes in cell cycle. OTA exposure led to the downregulation of *CDK2* expression (Figure 4A–C). The protein CDK2 works together with Cyclin E for proper G1/S transition. However, the latter was not found to be significantly regulated in our RNA-sequencing data.

To investigate its role in the OTA-induced phenotype, *CDK2* was overexpressed in HK-2 cells that were exposed to 0 or 100 nM OTA (Figure 4D), and subsequent cell-cycle analysis was performed (Figure 4E). The differences between the number of cells comprised in a cell-cycle phase when treated with 0 nM and 100 nM was used to quantify the OTA-induced effect. *CDK2* overexpression significantly reduced the effect induced by OTA (30 to 40% of the full effect) on the number of cells in G1 and S phases without fully preventing it. On the other hand, the OTA-induced effect on the number of cells in G2 phase was not influenced by *CDK2* overexpression. These results suggest that CDK2 may be involved in the OTA-induced G1 but not in G2 cell-cycle arrest.

### 3.6. E2F Is a Potential Master Regulator of the OTA Effect

**Figure 5:**
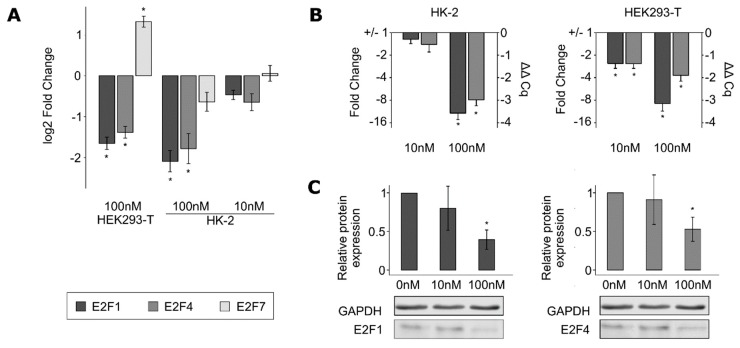
*E2F1* and *E2F4* were downregulated by OTA exposure. (**A**) *E2F1* and *E2F4* were significantly downregulated following 100 nM OTA exposure according to RNA-sequencing data in both cell lines, while E2F7 was found to be upregulated only in HEK293-T cells. Due to the consistent results between the two cell lines, the expression of *E2F1* and *E2F4* only was verified by RT-qPCR (**B**) and Western blot (**C**) (mean ± SD, * *p* < 0.05, *N* = 3).

In order to identify putative regulators of the genes comprised within the *brown* module (including *CDK2*), “upstream analysis” was performed with IPA Software on the list of genes belonging to this module. *E2F1* and *E2F4* were part of the top significantly enriched regulators (Table S5, Supplementary Materials). These genes encode for members of a transcription factors family involved in cell-cycle regulation [27,28]. *E2F1* and *E2F4* were found to be significantly downregulated in both HK-2 and HEK293-T cells after incubation with 100 nM of OTA in the RNA-sequencing data (Figure 5A), which could be confirmed by Western blot (Figure 5C). A significant downregulation was additionally observed from 10 nM OTA in HEK293-T cells while checking by RT-qPCR (Figure 5B). More generally, E2F-binding sites were significantly enriched in the promoter regions of the genes regulated by 100 nM OTA in both HK-2 and HEK293-T cells (Table S6, Supplementary Materials), suggesting that E2F transcription factors may be global regulators of the effect of OTA on gene expression.

## 4. Discussion

Transcriptome analysis of different human kidney cell lines after exposure to OTA was the strategy we adopted to elucidate mechanisms underlying the effect of this mycotoxin on human kidney. The results suggest that nanomolar concentrations of OTA lead to wide changes in gene expression in both HK-2 and HEK293-T cells, including for genes involved in pathways related to DNA damage and cell-cycle control. Moreover, OTA-induced cell-cycle arrests were observed in both cell lines.

In order to have a more stringent filter for genes regulated by OTA exposure, both DESeq2 and EdgeR [19,20] were used to perform differential expression analyses. Both tools estimate differential expression in different ways (dispersion parameters and type of normalization) and can, therefore, provide complementary information as previously discussed [29]. Moreover, working with two cell lines in parallel reduces putative cell-line-specific effects.

Previous studies showed interest in investigating the effect of OTA on the transcriptome of these cell lines. Global changes and regulation of cell cycle-related genes were also observed [30,31,32,33]. Unfortunately, most of these studies used OTA concentrations in the micromolar range (up to 500 times higher than the concentrations used in this study), which could partially explain the divergences between their results. For instance, one study reported that 16 µM OTA led to the downregulation of genes involved in cell-cycle progression in HEK-293 cells, but another one relayed that the exposure of HK-2 cells to 50 µM OTA led to the downregulation of genes involved in cell-cycle control, including *CDKN1A/p21*, which suggests it promoted cell-cycle advancement [31,32]. Similarly, effects of OTA on the cell cycle of kidney-derived cell lines were reported, and micromolar concentrations were associated with mitosis arrest in HEK293 and immortalized human kidney epithelial (IHKE) cells [34,35] and with S cell-cycle arrest in HEK293 [32,36]. On the contrary, we observed G1 and G2 cell-cycle arrests at 10 and 100 nM OTA, respectively. These variations between the studies support the approach of working with low concentrations of OTA in order to avoid unspecific toxicological effects.

A weighted network was built in an attempt to identify putative regulators of the OTA-induced cell-cycle arrests since using such analysis and, more specifically, identifying intramodular hubs were previously described as able to get gene lists with clearer biological association [25]. The weighted network generated here showed that *CDKN1A/p21*, which we firstly assumed as a key regulator of the OTA-induced cell-cycle dysregulation due to its strong regulation, is connected instead with genes related to cell death and survival. *CDKN1A/p21* and OTA were independently associated with these processes before [10,37,38]. On the other hand, the network analysis brought up cyclin-dependent kinase *CDK2* as a putative candidate for cell cycle regulation. The misleading first assumption regarding p21, based only on the regulation strength, underlines the necessity of using systematic data analysis methods combined with experimental validation in order to identify candidates for further investigations.

We report the downregulation of *CDK2* by OTA. Yang et al. previously suggested that the S cell-cycle arrest they observed was imputable to *CDK2* downregulation [36]. While *CDK2* necessity in G1/S checkpoint control and its possible compensation for by analogs such as *CDK1* were discussed [39,40,41], more recent results suggest that *CDK2* is actually required for properly timed cell-cycle progression [42], which implies that a downregulation of this protein would theoretically lead to an increased number of cells in the G1 phase. Additionally, *CDK2* was also proposed as an actor in G2 cell-cycle arrest after exposure to 2 µM OTA due to its role in cell senescence [43,44]. However, we observed that the G2 cell-cycle arrest induced by high concentrations of OTA was not influenced after *CDK2* overexpression, which goes against the idea of *CDK2* being a regulator of this process. However, *CDK2* overexpression was followed by a partial blockage of the OTA effect on the G1/S checkpoint. Although this limited effect may be due to reduced transfection efficiency or highlight that *CDK2* may act in concordance with other elements, these results bring up for the first time *CDK2* as a key regulator of the OTA-induced phenotype.

*CDK2* is regulated by different systems including by the p53/p21 pathway, and Yang et al. suggested that this pathway was responsible of OTA-induced senescence after exposure to sub-lethal concentrations still in the micromolar range [43]. However, we concluded that the p53/p21 pathway was not responsible of the changes in cell cycle induced by nanomolar OTA concentrations. On the other hand, while *CDK2* regulates *E2F* via its kinase activity (phosphorylates retinoblastoma Rb protein which releases *E2F*) and via direct interaction [45,46,47], it appears that at least *E2F4* may also be involved in its transcription regulation. Indeed, it was reported that knockdown of *E2F4* in intestinal cells is associated with decreased *CDK2* expression [48], and the promoter sequences of the latter contain conserved *E2F4*-binding sites [49]. This suggests that members of the *E2F* transcription factor family may be upstream regulators of the OTA-induced dysregulation of the cell cycle, which is supported by the fact that E2F-binding sites were enriched in the promoter regions of other OTA-regulated genes and that some *E2F* genes were downregulated by OTA exposure. Moreover, the differential expression analyses highlighted that OTA regulates protein-coding genes but also long non-coding RNAs. The latter play a role in gene expression regulation (as reviewed by Fernandes et al., 2019; Li et al., 2019 [50,51]) and are susceptible to environmental factors [52,53]. For instance, the transcript WISP1-AS1 was previously described as OTA-specifically induced and as involved in OTA-induced gene regulation and apoptosis [12]. In addition to providing a robust way of selecting relevant biological candidates, a network such as the one built in the present study also has the advantage of considering non-coding transcripts. Long non-coding RNAs downregulated by OTA were found strongly associated with *CDK2* in the *brown* module of the network, including some previously associated with cell apoptosis and oncogenic functions such as TP73-AS1, taurine upregulated gene 1 (*TUG1*), or X-inactive specific transcript (*XIST*) [54,55,56]. Based on the “guilt by association” principle, one can assume that these transcripts may have potential roles in cell-cycle regulation as well, in collaboration with or by regulating *CDK2*.

OTA was associated with chronic kidney damage [57,58], without identification of putative mechanisms. The cell-cycle arrest observed in the present study leads to a reduced regeneration capacity—which is normally high in proximal tubules—making renal tissue susceptible to normally harmless events (like exposure to drugs, toxins, or alterations in blood supply followed by reactive oxygen species (ROS) production). Thus, OTA seems to facilitate renal damage by lowering the threshold for pathogenic events.

In summary, the results of this first study integrating bioinformatics and classic biochemical approaches support that nanomolar concentrations of OTA lead to nephropathies by adjusting the expression of genes involved in cell-cycle regulation, which results in G1 and G2 cell-cycle arrests. We also propose that the OTA-induced G1 cell-cycle arrest is partially driven by CDK2, although the regulatory mechanisms of the latter remain to be clarified.

## Figures and Tables

**Table 1:** Genes regulated by 100 nM Ochratoxin A (OTA) in HEK293-T and HK-2 cells were enriched in DNA damage- and cell cycle-related pathways. Only the pathways having a significant adjusted *p*-value (−log(adjusted *p*-value) ≥ 1.3, equivalent to adjusted *p*-value ≤ 0.05) are displayed. The *Z*-scores correspond to the predicted regulation of the pathways (activation if Z > 0, inhibition if Z < 0), based on the log2 fold change of the genes enriched in the pathway (calculated by Ingenuity Pathway Analysis (IPA)). The *Z*-scores could not be computed for all pathways (lack of information concerning the impact of the regulation of some genes enriched in the concerned pathways). See also Figure S4 (Supplementary Materials).

IPA© Canonical Pathways	−log (Adjusted *p*-Value)	*Z*-Score
Nucleotide excision repair (NER) pathway	6.42	−1.789
Role of checkpoint (CHK) proteins in cell-cycle checkpoint control	2.49	−0.378
Role of breast cancer type 1 susceptibility (BRCA1) in DNA damage response	2.49	−0.378
Cell-cycle control of chromosomal replication	1.32	–
Hereditary breast cancer signaling	1.32	–

**Table 2:** OTA-regulated genes belong to groups of genes associated with cell cycle-related functions. Functional analysis was performed for all the modules with module Eigengenes highly correlated with OTA treatment (Figure 3A). The top five molecular and cellular functions groups identified by IPA are presented in this table. Each group contains several sub-functions (complete list in Table S7, Supplementary Materials) and the *p*-value ranges indicate the highest and lowest *p*-values obtained for these sub-functions. The last column contains the number of genes considered for each functional group.

Module	IPA© Molecular and Cellular Functions	*p*-Values Range	# Genes
*Black*	Cell death and survival	2.86 × 10^−2^–2.05 × 10^−6^	145
	RNA post-transcriptional modification	7.18 × 10^−3^–8.81 × 10^−6^	31
	Cellular assembly and organization	2.86 × 10^−2^–4.21 × 10^−5^	70
	Protein degradation	7.06 × 10^−3^–1.35 × 10^−4^	33
	Protein synthesis	2.28 × 10^−2^–1.35 × 10^−4^	52
*Brown*	Cellular assembly and organization	1.31 × 10^−4^–2.48 × 10^−8^	111
	Cellular function and maintenance	1.31 × 10^−4^–2.48 × 10^−8^	92
	RNA post-transcriptional modification	1.86 × 10^−5^–2.59 × 10^−7^	47
	Cell cycle	4.51 × 10^−4^–3.00 × 10^−7^	107
	Protein synthesis	2.33 × 10^−4^–1.08 × 10^−6^	80
*Green-yellow*	Cell cycle	4.06 × 10^−2^–2.14 × 10^−3^	14
	Post-translation modification	4.06 × 10^−2^–2.14 × 10^−3^	9
	Gene expression	3.06 × 10^−2^–3.33 × 10^−3^	39
	Cell morphology	4.06 × 10^−2^–3.41 × 10^−3^	14
	Cellular movement	4.06 × 10^−2^–3.41 × 10^−3^	12
*Pink*	DNA replication, recombination and repair	3.04 × 10^−3^–3.35 × 10^−8^	57
	Cell cycle	2.81 × 10^−3^–6.27 × 10^−8^	84
	Gene expression	1.08 × 10^−3^–9.40 × 10^−7^	95
	Cellular assembly and organization	2.03 × 10^−3^–1.72 × 10^−5^	80
	Cellular function and maintenance	8.52 × 10^−5^–1.72 × 10^−5^	53

## Supplementary Materials

The following are available online at https://www.mdpi.com/2073-4409/9/1/143/s1: Figure S1: The regulation of OTA of gene expression was determined using both EdgeR and DESeq2; Figure S2: OTA leads to the regulation of genes from different biotypes; Figure S3: Comparison of the differentially expressed genes identified in different experimental set-ups; Figure S4: OTA did not lead to changes in energy-related parameters; Figure S5: CDKN1A/p21 was upregulated by OTA but its clamping down did not restore the cell-cycle phenotype; Figure S6: Construction of a weighted correlation network in order to identify putative key drivers of the OTA-induced phenotype; Figure S7: Weighted correlation network analysis highlighted groups of genes correlated with OTA treatment; Table S1: Results of the differential expression analyses; Table S2: OTA influences a reduced number of genes in a dose-dependent manner; Table S3: Network analysis: genes in identified modules; Table S4: Functional analysis results; Table S5: Upstream analysis results for the *brown* module; Table S6: E2F-binding sites were enriched in promoter regions of OTA-regulated genes; Table S7: Buffer compositions.
