# Supplementary material for: Weighted Correlation Network Analysis Reveals CDK2 as a Regulator of a Ubiquitous Environmental Toxin-Induced Cell-Cycle Arrest
Source: Cells. 2020 Jan 7;9(1):143. doi: 10.3390/cells9010143 (PMC7017252; doi:10.3390/cells9010143)
Supplement: Supplementary file 1 [file cells-09-00143-s001.zip › Supplementary Figures.pdf]

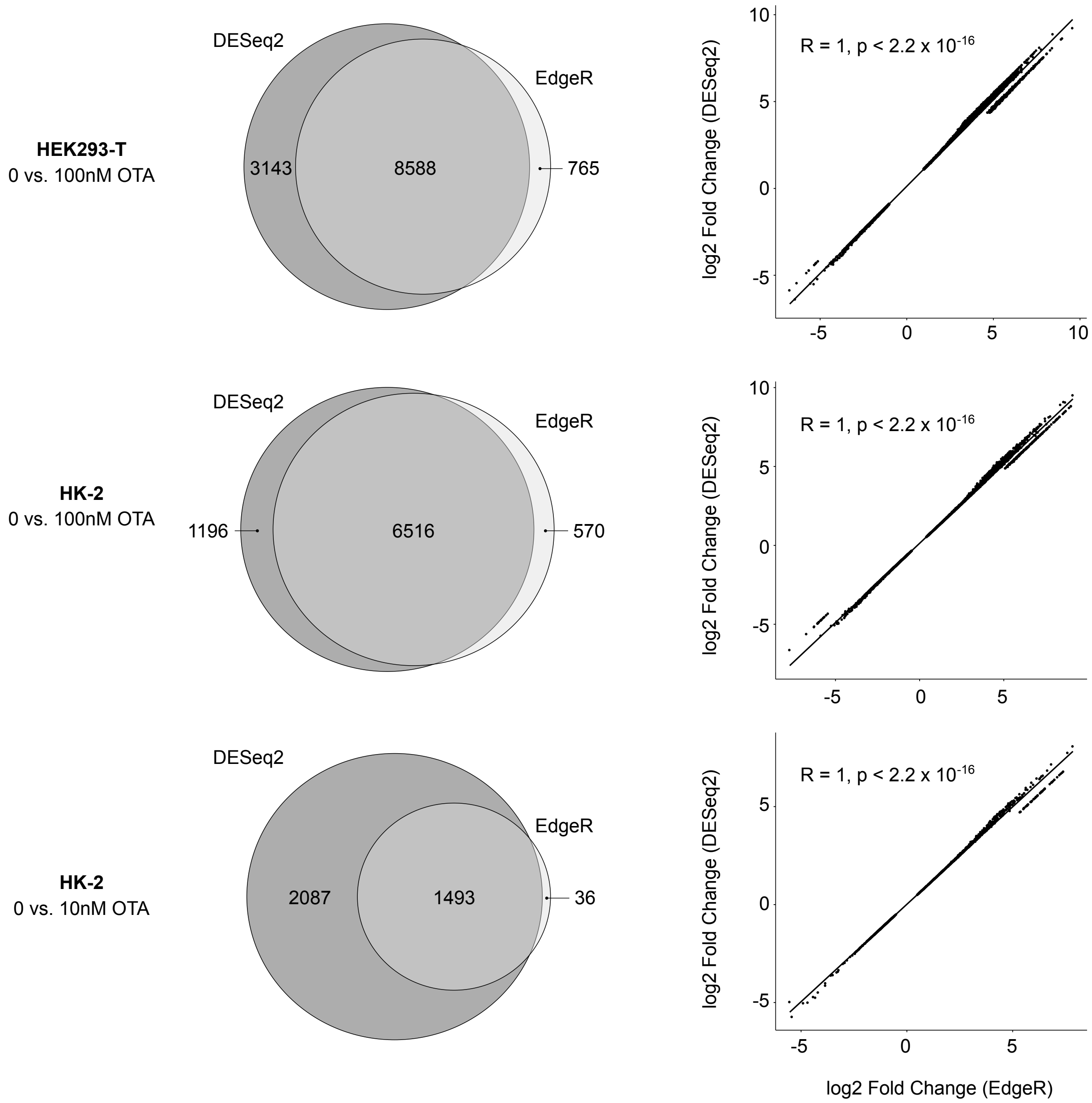

FigureS1.The regulation of OTA of gene expression was determined using both EdgeR and DESeq2.(Related to Figure 1) **(Left panel)** The list of genes defined as significantly differentially expressed (FDR 0.01) with both differential expression analysis tools were crossed for each condition. **(Right panel)** Comparison of the expression levels of differentially expressed genes (when compared to control) resulting from the analyses performed with EdgeR and DESeq2. Only the genes found to be regulated by OTA (FDR 0.01) are plotted.

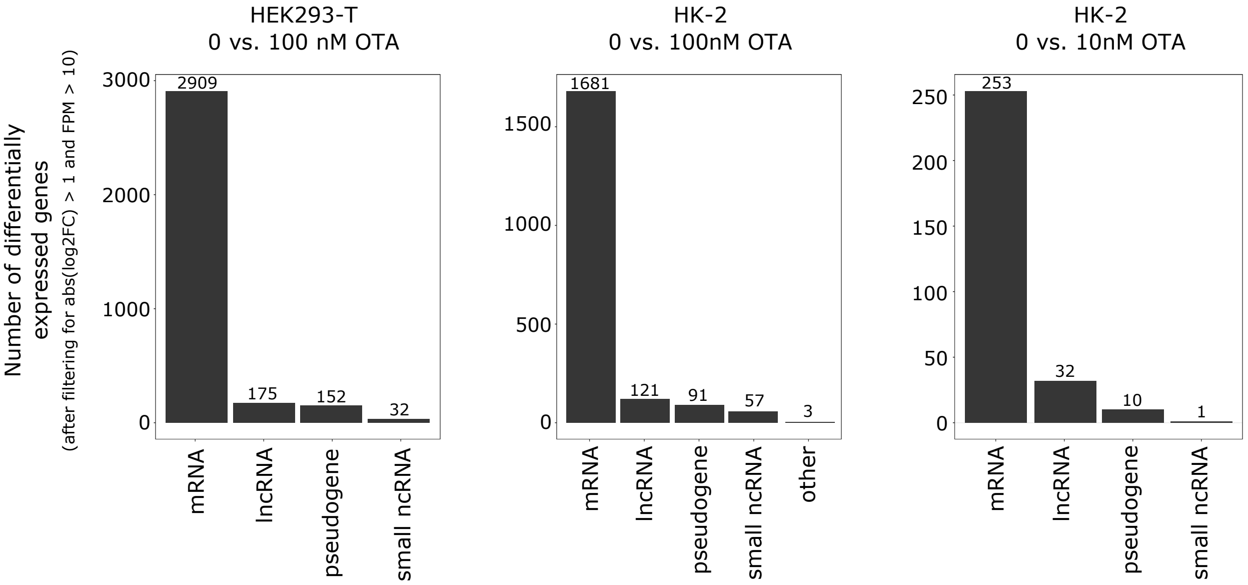

FigureS2.OTA leads to the regulation of genes from different biotypes. (Related to Figure 1) Biotypes were assigned according to the Ensembl annotation. Hence, "lncRNA" stands here for all non-coding transcripts longer than 200nt e.g. antisense, lincRNA, sens intronic, sense overlapping; "small ncRNA" stands for all small non-coding transcripts such as miRNA, scRNA, snoRNA; "others" stands for the transcripts that do not fit in the other categories (e.g. rRNA or tRNA).

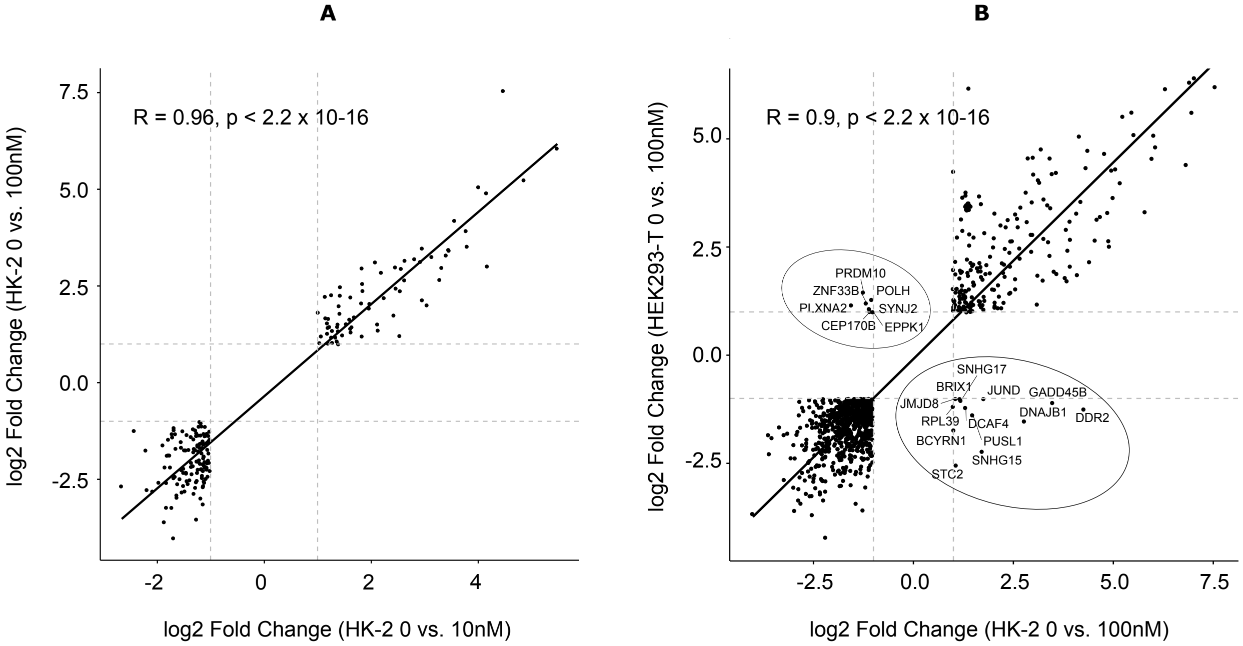

FigureS3.Comparison of the differentially expressed genes identified in different expreimental set-ups. (Related to Figure 1B) For each plot, only the genes found to be significantly regulated (FDR 0.01) and filtered (abs(log2 Fold Change) > 1 and FPM > 10) are plotted,hence the gaps between -1 and 1 (marked with dotted lines). The log2 Fold Change values result from the differential expression analysis with EdgeR. **(A)** Genes found to be differentially expressed in HK-2 cells after exposure to both 10 and 100 nM OTA. **(B)**Genes found to be differentially expressed after exposure to 100 nM OTA in both HK -2 and HEK293-T cells. Two groups of genes (identified by two ellipses) showed opposite regulation in the two cell lines

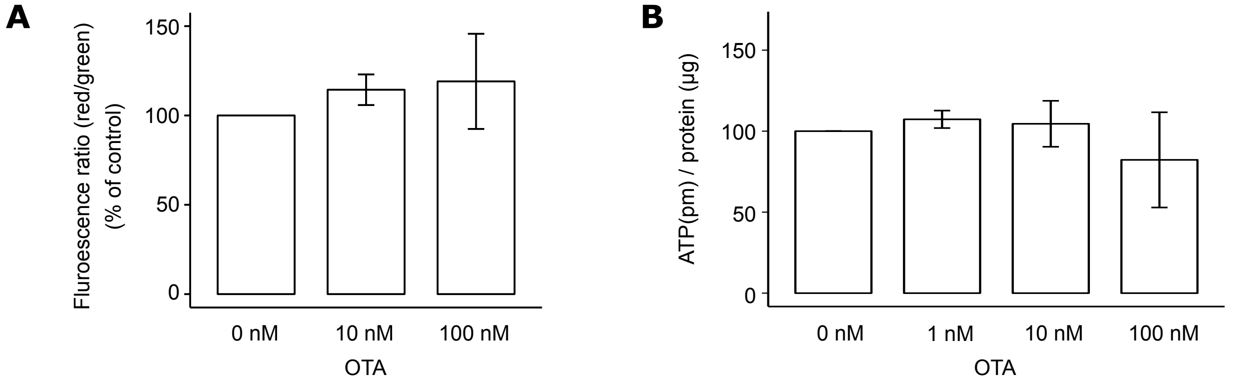

FigureS4.OTA did not lead to changes in energy-related parameters.(Related to Table 1) **(A)** The ratio of red and green fluorescences after incubation of the cells with JC-10 was used as a measure of the mitochondrial potential (correspond to "healthy" and "unhealthy" mitochondria, respectively). No significant differences were observed between the treatments. (mean ± SD, N = 3) **(B)** The amount of ATP (pm) was normalized to the protein amount (µg) for each measured well. No significant differences were observed between the treatments. (mean ± SD, N = 3)

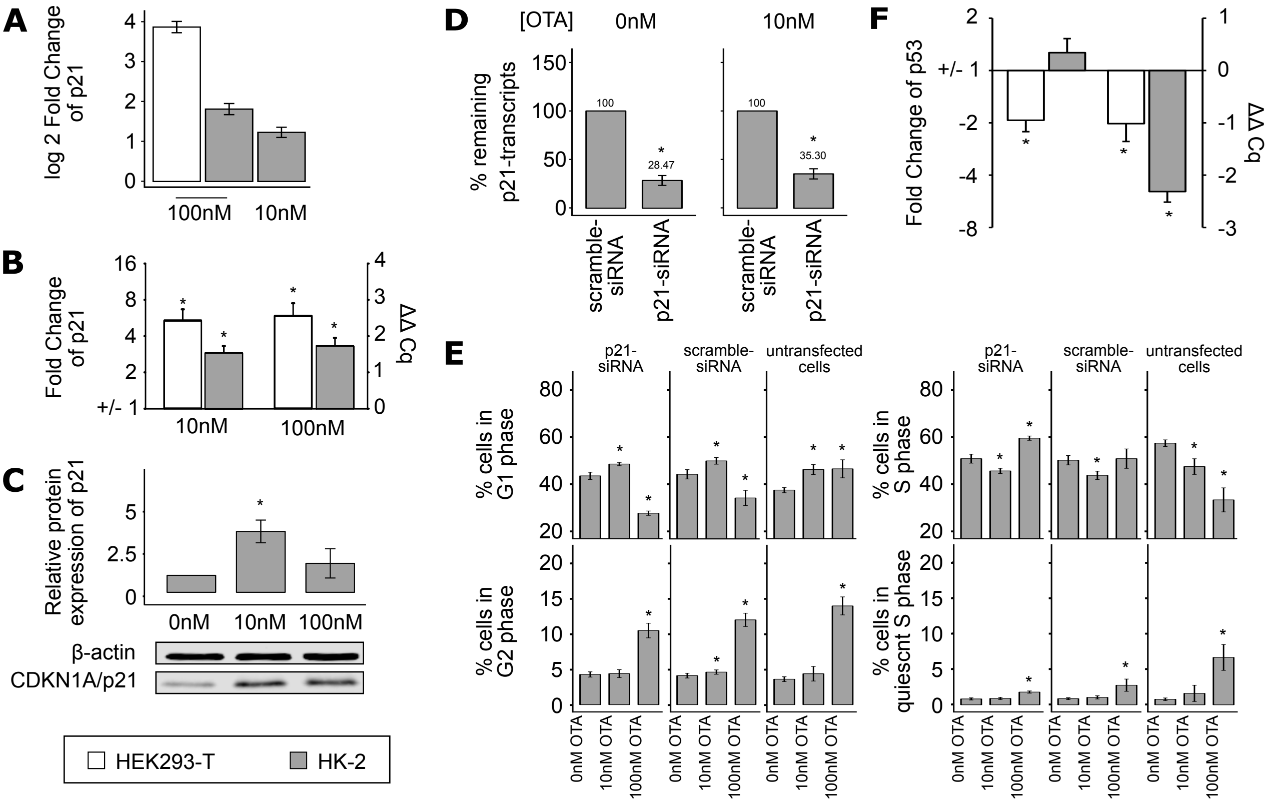

FigureS5.CDKN1A/p21 was up-regulated by OTAbut its clamping down did not restore the cell cycle phenotype.(Related to Figure 2)**(A)** CDKN1A/p21 was up-regulated following exposure to OTA according to the RNA-sequencing data. This could be confirmed by qPCR **(B)** and partially by WB **(C)**. **(D)** The expression of CDKN1A/p21 was clamped down using siRNA. **(E)** Cell cycle analysis was performed on cells transfected with p21-specific siRNA, with scramble siRNA and with untransfected cells. (mean ± SD, * $p < 0.05$, N = 3-4) **(F)** RT-qPCR showed that p53 was down-regulated by OTA-exposure. (mean ± SD,* $p < 0.05$, N = 3-4)

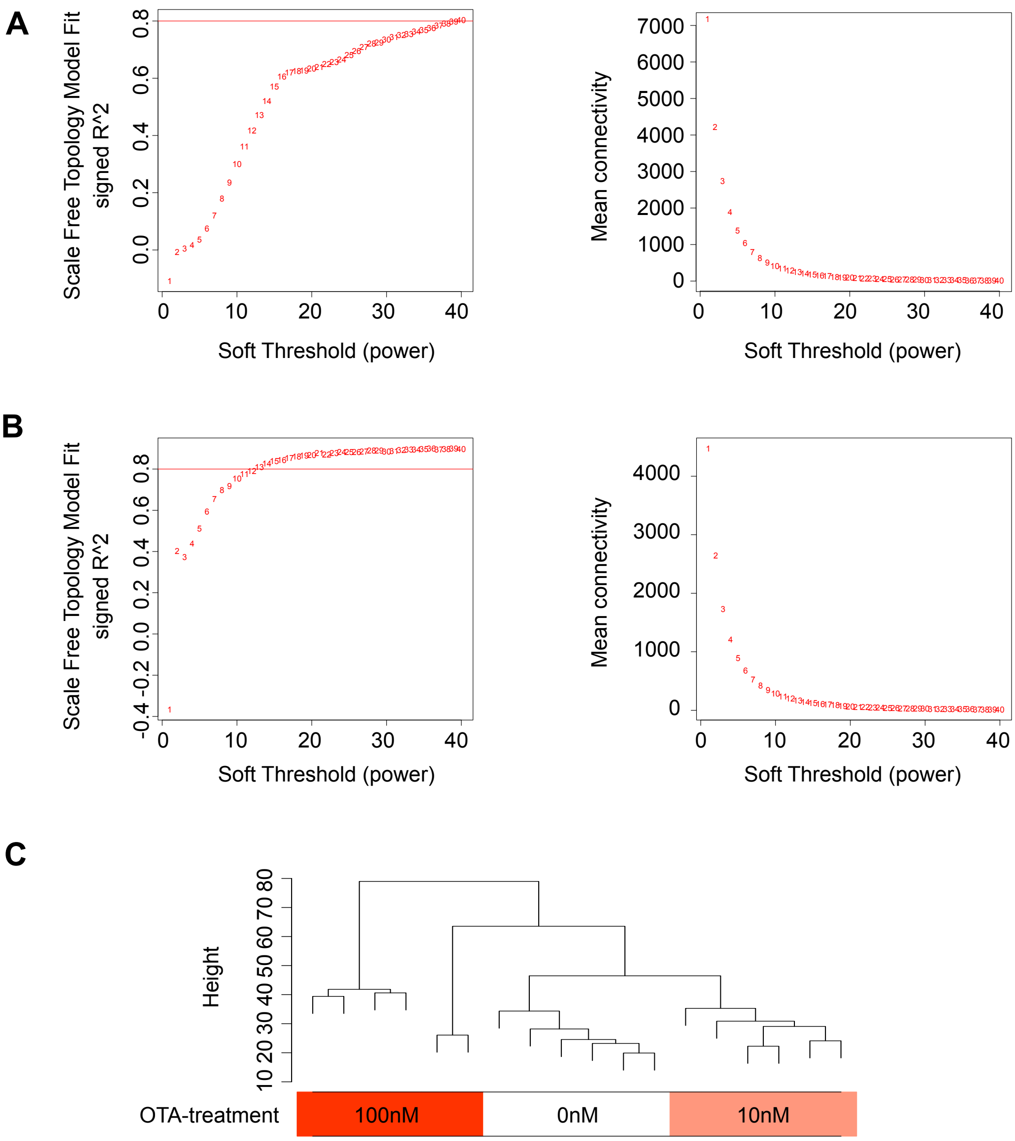

FigureS6.Construction of a weighted correlation network in order to identify putative key drivers of the OTA-induced phenotype.(Related to Figure 3)**(A)** A network was built with all the RNA-sequencing data available, from both HEK293-T and HK-2 cells. (Left) The linear regression model fitting index R2 was plotted for soft-powers β between 1 and 40. The horizontal line at 0.8 corresponds to the threshold to reach to have a network fulfilling the scale-free topology criterion. (Right) The Mean Connectivity was plotted for the same soft-powers β. **(B)** A second network was built on the data from HK-2 cells only. R2 was (Left) and the Mean Connectivity (Right) were plotted for soft-powers β between 1 and 40. **(C)** Clustering of the samples based on the normalized counts after filtering of the lowly expressed genes during the construction of the network with the data from HK-2 only (see (B)). Each branch corresponds to one sample.

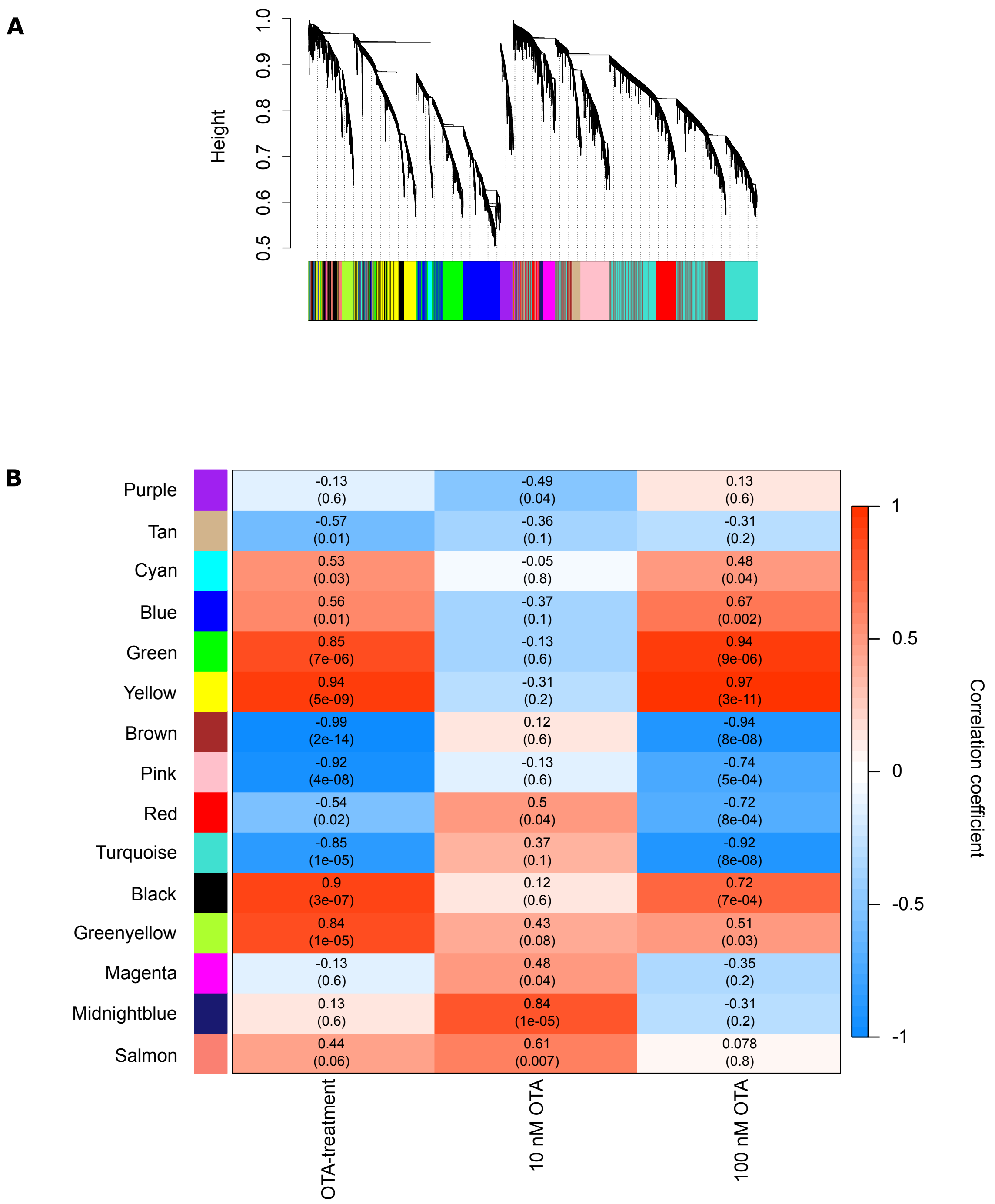

FigureS7.Weighted correlation network analysis highlighted groups of genes correlated with OTA-treatment. (Related to Figure 3) **(A)** Genes were clustered according to their dissimilarity. Each leave of the dendrogramm corresponds to a gene and the module to which this given genewas associated is represented by a color in the lower part. (B) The modules of genes defined by WGCNA are represented as a heatmap. The correlation between each Module Eigengene and the traits "OTA-treatment" (0, 10 or 100 nM OTA) and the two investigated concentrations of OTA (10 and 100 nM OTA) were calculated. In each case, the correlation coefficient and the corresponding p-value are indicated.
